# Direct Behavioral and Neurophysiological Evidence for Retronasal Olfaction in Mice

**DOI:** 10.1371/journal.pone.0117218

**Published:** 2015-02-12

**Authors:** Michelle R. Rebello, Padma Kandukuru, Justus V. Verhagen

**Affiliations:** The John B. Pierce Laboratory and Department of Neurobiology, Yale University School of Medicine, New Haven, Connecticut, United States of America; Technical University of Dresden Medical School, GERMANY

## Abstract

The neuroscience of flavor perception is hence becoming increasingly important to understand food flavor perception that guides food selection, ingestion and appreciation. We recently provided evidence that rats can use the retronasal mode of olfaction, an essential element of human flavor perception. We showed that in rats, like humans, odors can acquire a taste. We and others also defined how the input of the olfactory bulb (OB) -not functionally imageable in humans- codes retronasal smell in anesthetized rat. The powerful awake transgenic mouse, however, would be a valuable additional model in the study of flavor neuroscience. We used a go/no-go behavioral task to test the mouse's ability to detect and discriminate the retronasal odor amyl acetate. In this paradigm a tasteless aqueous odor solution was licked by water-restricted head-fixed mice from a lick spout. Orthonasal contamination was avoided. The retronasal odor was successfully discriminated by mice against pure distilled water in a concentration-dependent manner. Bulbectomy removed the mice's ability to discriminate the retronasal odor but not tastants. The OB showed robust optical calcium responses to retronasal odorants in these awake mice. These results suggest that mice, like rats, are capable of smelling retronasally. This direct neuro-behavioral evidence establishes the mouse as a useful additional animal model for flavor research.

## Introduction

Flavor perception is a multisensory experience provided by the convergence of sensory inputs such as taste, smell, touch, temperature, vision and hearing [1–5]. Retronasal olfaction plays a dominant role in flavor perception [6–8]. Aromas from food inside the mouth travel behind the palate and reach the nasal mucosa through nasopharynx. This is in contrast to orthonasal olfaction, perceived during inhalation or sniffing external odors via the nares [9,10]. The associated whole brain functional anatomy also differs [11–13]. Perceptual differences in relation to these odor routes, such as detection thresholds, intensities and odor identification have been observed and result in qualitatively distinct sensory experiences [9,11,12,14,15]. Perceptual integration with non-chemosensory stimuli also differs between the retro- and orthonasal routes [13,16]. The neural mechanisms underlying these differences remain largely unknown, but likely involve the insula, orbitofrontal cortex, amygdala and anterior cingulate cortex [12,16–19].

Understanding the neural basis of retronasal olfaction is important because the aroma released during the orosensory processing of food has great impact on appetite and satiety [20–23]. Studies have shown that food related odors induce salivation and release insulin and gastric acid [24]. Many diseases and medications affect the sense of smell, which also alters flavor perception, with the potential to aggravate the patient's health [25,26]. The importance of flavor on metabolic disease related to feeding has been clearly established [5].

Several human neuroimaging studies have explored the functional neuroanatomy of flavor perception (e.g. [1,2,12,13,27,28]). Despite this valuable progress, human neuroimaging is limited by spatial (mm) and temporal (seconds) resolution and cannot resolve the olfactory bulb (OB), the first relay in the olfactory system. We therefore established the rat model of flavor neuroscience to allow us to perform neurophysiological experiments that remain impossible in humans. We provided direct evidence that rats are capable of using the retronasal mode of olfaction [29]. Rats could detect tasteless licked retronasal amyl acetate down to 0.003% in water. We further showed that in rats odor-taste flavor percepts can develop in an experience-dependent way [30]. Having thus established the relevance of the rat to human flavor perception, we (for the first time in *any* species) defined OB retronasal responses in the anesthetized rat [31]. Lowe and colleagues subsequently characterized mouse OB retronasal responses [32], with results similar to that of the rat. Briefly, responses at the input of the OB were smaller to retronasal than orthonasal odors and the relative efficacy depended on the odors vapor pressure, but not polarity. By contrast, the relative dynamics were correlated with odor polarity, not vapor pressure [31]. These results explain for example the lower retronasal than orthonasal odor intensity in humans.

It is desirable to add the mouse as a model to study flavor neuroscience. Unlike the rat, the mouse provides useful transgenic approaches. These approaches include optogenetic control of [33], as well as endogenously encoded sensor-mediated report of neural activity of targeted neurons [32,34–36]. We hence sought to establish the relevance of mice to humans in food flavor perception by evaluating the ability of mice to perceive retronasal odors. Both to substantiate this and to illustrate the usefulness of transgenic mice we also optically imaged the OBs responses to retronasal odors in these awake mice.

We used GCaMP3-EMX and GCaMP2-OMP-tet mice as experimental animals. These animals express genetically encoded green fluorescence based calcium sensors in a subpopulation of inhibitory neurons (EMX [37]) and in olfactory receptor neurons (OMP) in the OB [38]. We used a direct behavioral approach to test the mouse's ability to detect and discriminate retronasal odors that were presented orally in aqueous solutions in a go no go task. The mice were presented with 3 odor concentrations in random order per day in which the mice had to choose the odor against deionized water. To validate that the mouse actually perceived the retronasal odors by olfaction alone, the olfactory bulbs were ablated and the task was repeated. We also performed calcium imaging of the dorsal olfactory bulb during odor presentation. Our findings support the relevance of mice as a model for flavor research to advance our understanding of the neural basis of flavor perception.

## Methods

### Subjects

Male GCAMP3-EMX1 (n = 4) and GCAMP2-OMP (n = 2) mice weighing 20–30 g were used in this study. In GCAMP3-EMX1, GCAMP3 is expressed in sub-populations of inhibitory interneurons in the OB [37], while GCAMP2 was expressed in the olfactory sensory neurons of GCAMP2-OMP mice. All mice were housed individually in an environment of controlled humidity (60%) and temperature (23°C). The vivarium was set with 12-h reverse light-dark cycles and all the behavioral training and experiments were carried out in the dark phase. Food was available *ad libitum* except during testing. Mice began water restriction at least 7 days post-surgery, and 3–4 days prior to behavioral training. During testing and training sessions, mice received approximately 2–3ml water. Data acquired from six mice are presented here.

### Ethics statement

All the animals were treated according to the guidelines established by the U. S. National Institutes of Health (1986), and the experimental protocols were approved by the Institutional Animal Care and Use Committee of the John B. Pierce Laboratory (Protocol 120).

### Surgical procedures

Mice were anaesthetized with ketamine and dexdormitor (75–100 mg/kg and 0.5 mg/kg respectively, i.p). Antisedan (0.5 mg/kg SC) was used for the reversal of the sedative effect. Toe-pinch reflex was checked before the start of the surgery as well as periodically during the surgery to ensure that the mouse was deeply anaesthetized. Throughout the surgery the mouse's core body temperature was maintained at 37°C with a thermostatically controlled heating pad (Omega Engineering Inc, Stamford, CT). The bone overlying the dorsal surface of the bulb was exposed, thinned and coated with cyanoacrylate glue to make the bone transparent. This yielded a ~10mm^2^ optical window which was clear for several months and was re-thinned when clarity was reduced. A custom made head-restraining cap (aluminum plate with 2 holes laterally for head-fixation) was attached to the exposed caudal skull using dental acrylic. Mice were allowed one week to recover before being put on water regulation in preparation for the start of training.

At the end of the study, a bulbectomy (OBx) was performed on the mice. Briefly, mice were anesthetized with Isoflurane (1.5–2.5%). A 2 mm diameter hole was drilled into the bone overlying the each dorsal OB. The OB was removed using vacuum suction and haemostatic gel foam was inserted into the cavity. The exposed area was covered with cyanoacrylate glue. Carprofen (5 mg/kg IM, Pfizer animal health, New York, NY) was also provided as an analgesic. After one week of recovery, mice were put on water restriction for 3–4 days and their performance was again tested on the go-no go task. This time the go-no go task also included sucrose as separate stimulus to ensure the mice could perform the task even if the bulbectomy prevented discrimination.

### Behavioral Training

**Fig 1:**
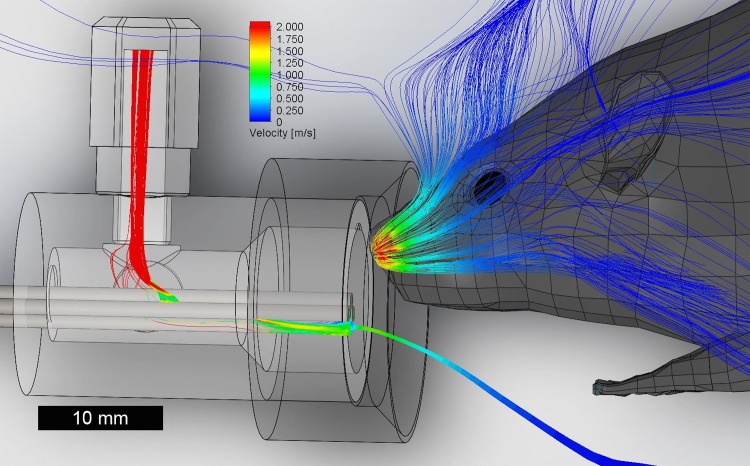
Flow modeling of the setup. A flow study was performed for particles at the tip of one of the lick spout tubes (in the center of the surrounding vacuum tube) and particles at the nares of a mouse model. The vacuum (upper left, red) generates an airflow preventing trajectories between the nares (inhaling, conservatively at the maximum rate reported for rats, red) and the lick spout.

Behavioral training for the go-no go odor discrimination task began by training animals to accept head fixation and to perform lick-no lick aqueous odor discriminations using water restriction for motivation. Initially mice were habituated to the head restraint and trained to lick for S+, after which they received a reward of approximately 8 μl of water. The S+ at the start of training consisted of a mixture of 0.1% amyl acetate and 3mM HCl, the latter added to provide a temporary taste guide during training [29]. The S- stimulus, deoxygenated deionized water, was introduced during the second day. An incorrect lick for the S- was punished with 8μl of 1M NaCl. The HCl taste cue was removed after a week of training. Subsequently the S+ was a pure odor solution. All training was performed in a custom restraint chamber (13.0 cm long x 3.8 cm wide x 3.5 cm tall, internally) made of transparent acrylic. A lick-spout was surrounded by a concentric vacuum tube and was positioned in front of the animal’s mouth for water reward and retronasal stimulus delivery (**Fig. 1**). The spout consisted of 9 23-gauge stainless steel tubes of 19 cm length, brazed together with flush cut tip. The inner surfaces of the chamber were cleaned with a moist paper towel between each behavioral daily session.

Each training session for a mouse lasted for approximately 30 mins and consisted of approximately 7–10 blocks, where a block consisted of 20 trials. The session was ended when the mice failed to lick during an entire block, which block was excluded from the data. S+ and S- trials were presented with a 50% probability during a trial. A single trial lasted for 12 s and was denoted by a trial start tone. A vacuum (1 s) at the start of the trial removed any solution adhering to the lick spout. The stimulus (8 μl) was presented 4 s into the trial, after which a second vacuum period (1 s) removed any residual S+ or S-. The reward/punishment was available 8s into the trial during which time the mouse had to decide whether to lick (S+) or withhold a lick (S-). In addition to the salt punishment, a loud tone also accompanied an incorrect lick. Six seconds separated trials from each other. The total number of correct responses (S+ lick or S- no lick) relative to the total trials comprised an index of behavior performance. A performance index of 40–60% was considered chance performance and a score of 100% was perfect discriminatory behavior. Signs of distress (vocalizations, defecation or failure to perform the task) resulted in cessation of the session and release of the animal from restraint. Odorant delivery, reward delivery, licking behavior and monitoring of performance were achieved through custom software written in LabVIEW (National Instruments, Austin, TX).

Three neighboring (1 or 2 log units spaced) concentrations of AA were presented during each session through dedicated lick spout tubes in random order. Upon reaching performance criterion for all three levels during a session the concentration was lowered 10-fold during the next session. Each concentration was tested on at least 2, but typically 8 sessions (8.1±5.3, mean±sd). Before starting randomized S+ stimulus presentation on a given session, mice were tasked to discriminate the lowest concentration of AA they were able to discriminate at the prior session. Once they were able to discriminate this stimulus with an accuracy of >70% the randomized presentation of the thee S+ odorants started for the remainder of the session. This ensured that they could perform the task. Mice performed 26±3 trials before randomization started. Performance data were based on the average performance across these randomized trials. The last trials of the last block that consecutively showed no responses (satiation) were omitted.

### Gustometry

The 8-channel gustometer assembly served for the delivery of retronasal aqueous odor stimuli, as well as the water reward through dedicated channels for each solution. The gustometer consisted of 1 or 5 L glass bottles, Teflon tubing, connectors and manifold. One of the channels was used for rinsing the lick manifold after each stimulus delivery; another one was used to vacuum the manifold during and after rinsing. Odorants, with or without tastants, were dissolved in distilled water. Nitrogen (2.5 psi) was used to pressurize the fluids and to prevent oxidation of the odorants in deoxygenated deionized water. Three valves each were used for S+ and S- stimuli and randomization was enabled for both. Sound masking was enabled such that whenever S+ or S- valve opened during a trial, 8 other dummy valves also clicked open, obfuscating any sound cues arising from the of S+ or S- valves. A continuous vacuum sucking air (10 L/min) from around the lick spout was used to prevent orthonasal contamination, confirmed by 3D flow models (**Fig. 1**). Licking was detected using a capacitative lickometer (Med Associates).

### Flow modeling of retronasal setup

In absence of mouse sniff volume flow data we conservatively used rat data. Rats can sniff up to 19ml/s maximally (Youngentob et al.), 11% of our lick-spout vacuum flow rate of 167ml/s. Nevertheless, to ensure mice could not detect odorants licked from the lick spout we modeled the setup in 3D in SolidWorks 2013–2014 and Flow Simulation 2013. A rat 3D model (www.3dcadbrowser.com, model 4621) was scaled down to real mouse dimensions, placed centered in front of the lick spout closely (mm-level accuracy) matching the real setup. It was meshed with 315,251 fluid cells and 400,372 partial cells in a 6.8 L box (**Fig. 1**). A uniform initial mesh was used (Mesh level 3). Both nares were modeled as an Outlet Volume Flow (1.8*10^-5^ m^3^/s each, the maximum total sniff flow rate reported for rats [39]). The box boundary was set as Static Pressure (101,325 Pa) and temperature at 20°C. The lickspout vacuum tube connector was modeled as an Outlet Volume Flow (1.6*10^-4^ m^3^/s). Solutions of steady state flow were reached after ~600 iterations which required ~3 hrs. time on an 8- core (Core i-7) PC.

### Odorants and tastants

A variety of monomolecular odorants was selected based on their solubility and previous use in olfactory research involving rats. All odorants (amyl acetate (AA), methyl valerate (MV), ethyl butyrate (EB)) and tastants (HCl, sucrose) were reagent grade and purchased from Sigma (Sigma-Aldrich, St. Louis, MO). Odorants were stored in the dark under nitrogen. Only AA was tested behaviorally, all three odors were tested using optical calcium imaging.

### Optical imaging

Optical signals were recorded using a CCD camera (Redshirt Imaging, LLC) with 256 x 256 pixel resolution, and at a frame rate of 50 Hz. This pixel resolution is sufficient to resolve single glomeruli at magnifications low enough to image across the entire dorsal surface of the bulb. The epifluorescence microscope is a custom made tandem-lens type [40] with high NA (0.85–0.95) CCTV objectives for high SNR. It is accurately adjustable in height and along one angle to allow optimally focused and reproducible recordings. A high-power LED (Luxeon) driven by a DC power supply acted as the light-source. A DC amplifier powered a peltier device onto which the LED was glued. The LED-cooling peltier current was proportional to the LED current, yielding a stable illumination. The fluorescence filter set used was BL P01-514 (excitation filter), LP515 (dichroic), and LP530 (emission filter; Semrock, Lake Forest, IL, USA). Raw images were converted to images representing the relative change in fluorescence (%ΔF/F) in each pixel and frame after stimulus application. Functional images were low-pass filtered using a Gaussian function with 5-pixel basis. Temporal traces were band-pass filtered (0.2–4Hz, Butterworth). Data analysis was performed using NeuroPlex software (RedShirtImaging LLC, GA, USA).

### Data analysis

The percentage of correct licks (for S+) and the correct rejections (for S-) across all trials for each stimulus were averaged to obtain a daily % correct per stimulus per animal. The final % correct per stimulus condition was obtained by averaging across daily % correct and subsequently across animals. These data were further analyzed by analysis of variance (ANOVA) (test stimulus as main factor) and planned t-tests. All analyses were performed in Microsoft Excel 2010. Averages are reported ± sem. Alpha level was set at 0.05. Behavioral performance data are available from the Dryad Digital Repository: http://doi.org/10.5061/dryad.3cs6v.

## Results

### Mice can detect retronasal odorants

A cohort of 6 water-restricted mice was trained to detect retronasal odors in a go-no go task. Initially to aid in the training, a taste cue (3mM HCl) was provided along with the S+ aqueous retronasal odor solution (0.1%) amyl acetate. Amyl acetate was chosen as it is known to not have an orosensory component at concentrations 0.1% and below in rats [41]. Orthonasal contamination was removed by the presence of a vacuum flow around the lick spout, which prevented any odorant from reaching the nose as shown by 3D flow modeling (**Fig. 1**).

**Fig 2:**
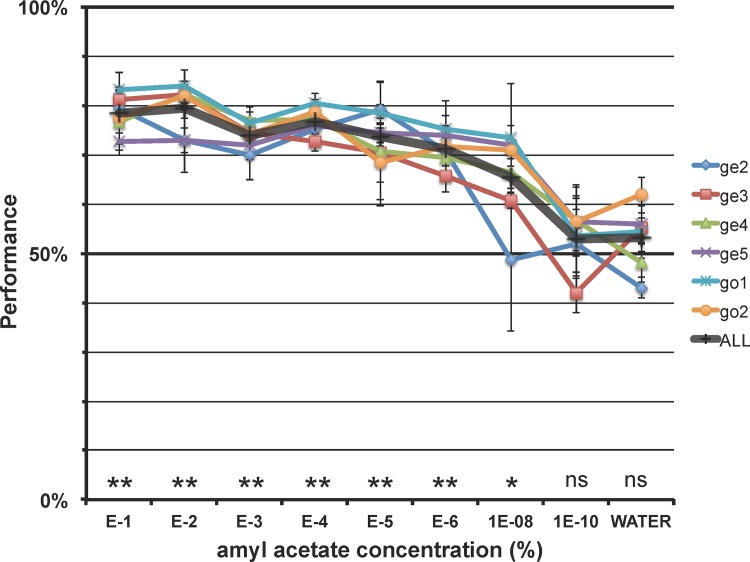
Retronasal odor discrimination performance of mice. Graph showing average (±SEM) and individual performance accuracy of 6 mice discriminating between a range of concentrations of licked retronasal amyl acetate (AA) and water. ** p< 10^-7^, * p<0.0002, ns not significant (unpaired t-test for performance >50%). Mouse labels: ge: GCaMP3-EMX; go: GCaMP2-OMP.

After mice successfully discriminated between water (S-) and the S+, the HCl taste guidance was removed. Subsequently, the detection relied solely on the retronasal odor. We randomly switched between 3 channels of the gustometer for both S+ and S-, to ensure that mice were not able to use any possible cue associated with a particular valve. Mice were able to discriminate between water and 0.1% AA with an average accuracy of 78±2% (mean±SEM, p<10^-7^ above 50% (unpaired 1-tailed t-test), n = 6, **Fig. 2**). We next sought to determine the retronasal detection threshold for amyl acetate. Mice were found to successfully detect down to 10^-6^% AA (71±1% accurate, p<10^-7^) and 10^-8^% AA (65±4%, p<0.002), below which concentration their accuracy was 53±2% (10^-8^%) and 53±3% (water S+ controls) and did not differ from chance (**Fig. 2**). Performance on all but 10^-10^% AA was significantly above water controls (p<0.005, 1-sided paired t-test). ANOVA showed a significant effect of concentration of performance (p < 10^-6^).

### Bulbectomies prevent odor but not taste discrimination

**Fig 3:**
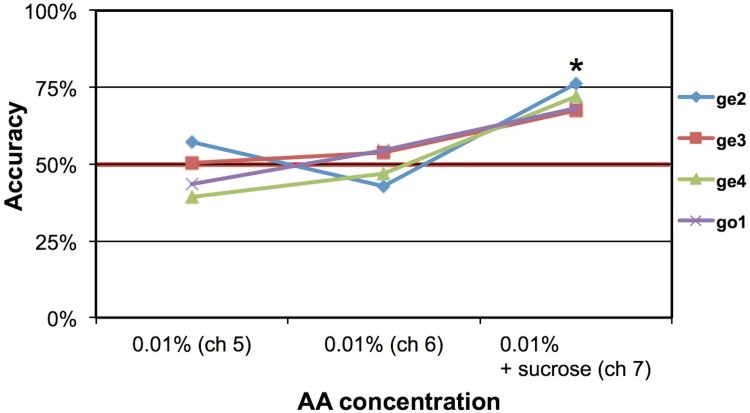
Accuracy after bulbectomy of 4 mice. Bulbectomized mice were unable to perform the discrimination task on two S+ gustometer channels presenting only the odor, but could detect the stimulus when admixed with 0.1M sucrose. * p<0.0001 (unpaired t-test for performance >50%). Mouse labels: ge: GCaMP3-EMX; go: GCaMP2-OMP.

To ensure that discrimination was not due to any possible taste component bulbectomies were performed on the 4 remaining mice at the end of the study (2 died after OBx). Mice could not perform the retronasal odor discrimination task accurately (48±6%, n.s. (n = 8: 4 mice x 2 channels) (**Fig. 3**). They were however able to discriminate the sucrose presented via a third channel (71±4%, p<10^-4^). We therefore confirm that odor detection occurred solely on the basis of retronasal smell.

### Optical imaging of retronasal responses in the dorsal OB

To confirm the behavioral evidence for retronasal odor detection we looked for evidence of bulbar retronasal responses. During one session the dorsal OB of the GCAMP3-EMX1 mice was imaged while they performed the retronasal go-no go task. We imaged one OMP mouse on two session, but this data yielded insufficient signal to noise ratio (data not shown). We used 0.67% ethyl butyrate (EB) and 0.54% methyl valerate (MV) aqueous solutions as the S+ and water as S-. These stimuli and their comparatively high concentrations were chosen to maximize retronasal OB response magnitudes.

**Fig 4:**
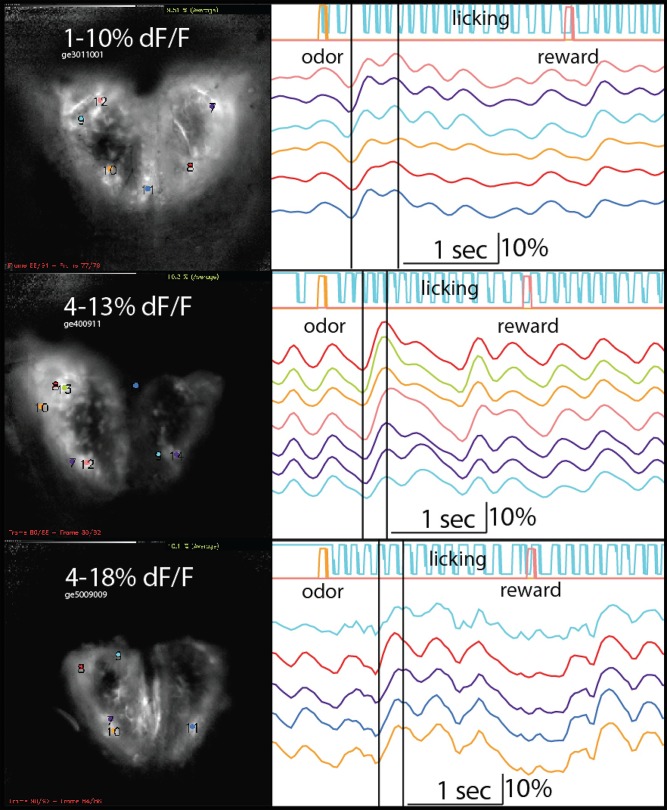
Optical imaging of retronasal OB responses. One trial of OB retronasal responses to 0.67% EB is shown for each of 3 GCAMP3-EMX mice (ge3, 4 and 5). Left: evoked maps and ROIs. Grey-scaling was applied between indicated minimum and maximum %ΔF/F. The OBs rostro-caudal orientation is bottom-top. Right: time-traces of ROIs, licking (blue), licked S+ odor valve open time (yellow) and water reward valve open time (pink). The two vertical lines indicate the center of the reference frames and response frames on which the response maps are based. Calibration bar: 10% ΔF/F.

Fig. 4 shows example trials for 3 mice. Fluorescence is modulated throughout the trials by sniffing, as shown previously in rats [42,43] (sniffing was unfortunately not measured due to technical issues). Licking (blue) did not directly modulate the activity in the bulb. Odor presentation at the lickspout is indicated by the yellow pulse at the beginning of the trials. Odor responses (that enhanced the sniff-modulated signal amplitude) of 10–18% ΔF/F were seen within the first 1, 2 or 3 licks (**Fig. 4, top to bottom: mouse ge3, ge4 and ge5)** of the retronasal odorant (**Fig. 4, right**). Response magnitudes (dF/F at the right vertical line minus the left line in Fig. 4) were 8.5±0.4%, 10.6±1.9% and 10.0±0.7% dF/F across the indicated glomeruli of the three mice, which were significantly higher than the pre-odor sniff-modulated response magnitude of the preceding peak (3.0±0.2%, 6.2±0.4% and 4.3±0.8%, respectively; P<10^-4^, 10^-2^, and 10^-3^, respectively, 1-sided paired t-test). On average the odor response was 2.2 times larger than pre-odor sniff-modulated signal. Response maps showed clearly visible glomeruli (**Fig. 4, left**). The response map of ge4 was mostly on the right side. The response amplitudes subsequently tended to decrease (ge3, ge4), but could also be sustained (ge5). In some cases (ge3, ge5) responses again increased after 1 and 4 licks, respectively, after presentation of the water reward (pink).

## Discussion

In this study we tested the hypothesis that mice, like rats and humans, can detect odors retronasally. We trained transgenic head-fixed mice to perform a taste-discrimination task after which the tastant was removed (as before [29]). We also assessed if retronasal responses could be observed in the dorsal OB of the same mice. Our results unambiguously support the hypothesis: they were able to detect the odors significantly above chance (**Fig. 2**). Further, we found clear evidence for bulbar responses (**Fig. 4**).

Mice on average were found to be 3 orders of magnitude more sensitive to retronasal AA compared to rats, which we reported to show a detection threshold of 0.003% AA [29]. However, the test procedures were not identical between these two studies. While the rats were presented with only a single concentration per session, in the current study the mice were tested on 3 neighboring concentrations at the same day. We chose the latter approach to present the mice at all sessions with at least one odorant they were known to be able to detect (on the previous session) and hence remain task-engaged and maximize the sensitivity of the detection threshold determination. It is hence entirely possible that the rat retronasal AA detection threshold does not differ from that of mice. Either way, both species are now known to be able to detect retronasal tasteless odorants down to very low ppm-ppb concentrations in water.

We controlled for three potential confounds: gustometer cues, non-olfactory orosensory cues and orthonasal cues. We avoided gustometer-related cues such as audible valve clicking or non-auditory vibrations by always co-activating a number of valves among which the stimulus valves were spatially located. Thus, cues pertaining to both distinct stimulus valve vibrations and their location were masked. Non-olfactory orosensory cues were avoided by choosing an odorant shown to be only detectable via smell in rats [41]. This was verified by our OBx control experiment to be true for mice as well (**Fig. 3**). Last, orthonasal cues were avoided by using a vacuum flow around the lick spout. Flow modeling showed that the mice would be unable to smell an odor orthonasally from the lickspout even when provided with a worst-case sniff-flow ability of rats (**Fig. 1**).

The bulbar responses to licked odorants (**Fig. 4**) are the first demonstration of retronasal odor responses in awake mammals. They highlight that during food ingestion the timing of the retronasal responses depend not only the intake of the food, but presumably also on the time of swallowing and sniffing (neither of which we were able to measure in these sessions). As multimodal integration of orosensory and olfactory stimuli depends on temporal congruence [1,2,4,5,8,44], these variations may have significant consequences for the establishment of flavor percepts. They further show a large variability of responses across subsequent sniffs (for some trials they linger while for others they decrease) and intake of unodorized water (for some this again evoked OB responses). Thus, retronasal smell during food intake is a complex phenomenon under control of multiple behaviors (licking, swallowing and sniffing).

The examples from our optical imaging experiments shown here illustrate the power of the transgenic model. We have made many (>10 rats) attempts at imaging retronasal responses in awake rats infused with calcium dye, but never were convinced these yielded clear evidence of retronasal responses. The results from these GCaMP3-EMX mice far exceed the signal to noise from the rat experiments. The advantage of the EMX driver line is that it expresses in a large population of neurons thereby yielding very high signal to noise ratios. We are hence finally in a position to explore retronasal neural responses in the awake mouse model of flavor neuroscience. The advantages of the mouse over the rat model are clear: they provide endogenously encoded calcium (used here) and voltage dyes in targeted neurons [34–36,45], as well as optogenetic control of specific neuronal populations [33]. These transgenic approaches will allow for specific inquires of the neural circuitry and behavior pertaining to food flavor perception.
